# The mediating role of sarcopenia in the link between loneliness and frailty among nursing home residents: a cross-sectional study

**DOI:** 10.1186/s12877-025-06342-5

**Published:** 2025-09-26

**Authors:** Zhenfei Chen, Dongxing Zheng, Shangqing Wu, Jiaze Dai, Haiman Huang, Yu Yang

**Affiliations:** 1 Department of Geriatrics, Affiliated Hospital of Guangdong Medical University, zhanjiang, Guangdong China; 2 Medical laboratory Center, Affiliated Hospital of Guangdong Medical University, Zhanjiang, Guangdong China

**Keywords:** Sarcopenia, Nursing homes, Elderly, Loneliness, Frailty

## Abstract

**Background:**

The present work focused on investigating the frailty status of older nursing home residents by analyzing the relationships among loneliness, sarcopenia, and frailty. The study explored the mediating effect of sarcopenia on loneliness and frailty among elderly individuals.

**Methods:**

From January to June 2022, 190 elderly individuals were enrolled from four nursing homes in Guangzhou, Dongguan, Zhanjiang, and Nanning using a convenience sampling method. A field survey was conducted using a sociodemographic questionnaire, the Frailty Phenotype, the UCLA Loneliness Scale, and the SARC-F screening tool. The data were analyzed with SPSS 25.0 and Process 4.1 software. Normally distributed continuous variables are presented as the means ± standard deviations (Mean±SD) . Associations among loneliness, sarcopenia, and frailty were assessed via Pearson correlation analysis. Moreover, the mediating effect of sarcopenia on loneliness and frailty was examined via linear regression. The bootstrap method in Process 4.1 was employed to test its mediating effect.

**Results:**

The frailty prevalence among elderly nursing home residents was 34.74%. The loneliness score was positively related to the frailty score (*r* = 0.156, *P* < 0.05, 95% CI:0.014–0.292), the sarcopenia score was positively related to the frailty score (*r* = 0.512, *P* < 0.01, 95% CI:0.399–0.610), and the loneliness score was positively related to the sarcopenia score (*r* = 0.214, *P* < 0.01, 95%CI:0.074–0.346). Sarcopenia demonstrated a complete mediating effect on loneliness and frailty among elderly nursing home residents. Sarcopenia typically achieved a mediating effect of 0.107 (95% CI: 0.026–0.197), accounting for 68.59% of the overall effect.

**Conclusions:**

Sarcopenia is a key factor influencing the occurrence of frailty in lonely elderly individuals in nursing homes.

**Supplementary Information:**

The online version contains supplementary material available at 10.1186/s12877-025-06342-5.

## Background

As the global population continues to age rapidly, traditional home-based elderly care is encountering considerable challenges, leading an increasing number of older adults to opt for residence in nursing homes. These facilities provide not only medical services and daily living support but also play a crucial role in facilitating social interaction and offering psychological support. Nevertheless, elderly residents in nursing homes often face specific health concerns, with loneliness and frailty being particularly prevalent [1]. Frailty is commonly observed among older individuals and is characterized by a decline in physiological reserve capacity. This condition is associated with an elevated risk of disease, reduced resilience to stressors, and decreased ability to maintain physiological homeostasis [2]. Studies have shown that in comparison with those in community-dwelling elderly individuals, frailty levels are greater in elderly nursing home residents [3]. Frail elderly individuals are a vulnerable group that faces complex health issues. Previous studies have indicated that frail elderly individuals have an increased risk of adverse events, such as disability, falls, hospitalization, or even death [4]. Frailty can be reversible, but actively identifying related risk factors is crucial for preventing, delaying, and reversing its progression [5].

Loneliness is an unpleasant feeling caused by differences in individuals’ expected and actual levels of socially meaningful relationships [6]. According to statistics, the incidence of moderate loneliness among elderly individuals in nursing homes is 61%, whereas that of severe loneliness is 35% [7]. Studies have shown that loneliness is closely linked to frailty. It can lead to reduced physical activity, sleep disorders, and poor dietary habits in older adults, thereby triggering adverse health conditions and accelerating physical decline [8, 9]. Studies have indicated a correlation between loneliness and sarcopenia [10]. Sarcopenia, a progressive systemic skeletal muscle disease, is characterized by the progressive deterioration of muscle mass, strength and function with age [11]. According to recent studies, the incidence of sarcopenia is about 10% in individuals aged ≥ 60 years [12]. Although classified differently, sarcopenia and frailty share many clinical similarities, suggesting that sarcopenia may be a potential mechanism linking loneliness to frailty in elderly individuals. Therefore, this study aims to explore the mediating role of sarcopenia in the relationship between loneliness and frailty in nursing home-based elderly individuals, providing scientific evidence for frailty prevention and treatment in this population.

## Methods

### Study sample

From January to June 2022, a total of 190 elderly individuals were selected through convenience sampling from four nursing homes located in Guangzhou, Dongguan, Zhanjiang, and Nanning, within Guangdong and Guangxi Provinces. Using G*Power software, the parameters were set as follows: medium effect size (f^2^ = 0.15), statistical power (1 - β = 0.80), 12 predictor variables, and a significance level (α = 0.05). Accounting for an anticipated 10% dropout rate, the minimum required sample size was calculated to be 142. A total of 190 valid questionnaires were ultimately collected, satisfying the sample size requirement. The inclusion criteria were as follows: ① were ≥ 60 years old; ② had resided in a nursing home for more than three months; and ③ were willing to take part in this research and actively cooperate.The exclusion criteria were as follows: ① severe diseases such as myocardial infarction, heart failure, chronic obstructive pulmonary disease acute exacerbation, stroke, or uremia; ② terminal stage (life expectancy ≤ 6 months); ③ cognitive impairment; and ④ hearing or visual impairments. The present work was approved by the ethics committee of the Affiliated Hospital of Guangdong Medical University (approval number:PJ2021–136). All the elderly participants voluntarily signed informed consent forms.

### Measurement instruments

#### Sociodemographic questionnaire

The sociodemographic questionnaire covered age, sex, height, weight, marital status, education, monthly income, disease type, walking assistance, and leisure activities.

#### Frailty phenotype

The Frailty Phenotype Scale, developed by Fried et al. [2], was used to assess individuals’ physical frailty status. The scale comprises five items: involuntary weight loss, slowed walking speed, grip strength decline, reduced physical activity, and self-reported exhaustion. Each item is scored as 1 if met and 0 otherwise, with total scores ranging from 0 to 5. Specifically, scores ≥ 3, 1–2, and 0 suggest frailty, prefrailty, and non-frailty, respectively.

#### UCLA loneliness scale

This scale was proposed by Russell et al. [13]. Studies have shown that Cronbach’s α coefficient of its Chinese version is 0.92 [14]. In this study, this scale was used to assess elderly individuals’ loneliness levels. It covers 20 items, with 11 positively worded items and 9 reverse-scored items. The 11 positively worded items focus on loneliness on a 4- to 1-point scale (always to never). The 9 reverse-scored items also focus on loneliness but use a 1- to 4-point scale (always to never). The total score ranges from 20 to 80, with greater scores indicating more severe loneliness (20–34, 50–80, 35–49: low, moderate, and severe loneliness, respectively).

#### SARC-F screening tool

The SARC-F questionnaire was developed by the British researcher Dr. J.E. Morley [15]. Studies have shown that Cronbach’s α of its Chinese version is 0.73, with item-total correlations all above 0.4 [16]. This scale was employed in the present work to screen for sarcopenia. The scale consists of five items that analyze an individual’s strength, ability to walk, ability to rise in a chair, ability to stair and fall. This questionnaire uses a three-point scoring system (0, 1, 2 points). Its total score ranges from 0 to 10, with greater scores indicating severe sarcopenia (0–3 points and ≥ 4 points: not having and having sarcopenia, respectively).

### Data collection

Data for this study were collected on-site by trained researchers. Prior to the survey, participants were informed of the study’s purpose and provided written informed consent. Standardized instructions were employed to explain each item to the participants, and responses were recorded accordingly. For gait speed measurement, participants were instructed to walk a straight-line distance of 4.5 m, and the time taken to complete the distance was recorded. Grip strength was assessed using an electronic dynamometer (CAMRY, EH101), with each hand measured once; the highest value of the two measurements was recorded as the final grip strength. The questionnaire survey was conducted within a controlled duration of 20 to 30 min. Upon completion, each questionnaire was reviewed and verified to ensure completeness and accuracy. This study distributed 210 questionnaires, excluded 20 invalid questionnaires, and obtained 190 valid responses, with a valid return rate of 90.5%.

### Data analysis

In this study, the data were analyzed with SPSS 25.0 software. Categorical variables are represented by frequencies and percentages, whereas normally distributed continuous variables are represented by means ± standard deviations (Mean±SD). Associations among loneliness, sarcopenia, and frailty were examined via Pearson correlation analysis. The potential mediating effect of sarcopenia on loneliness and frailty was tested via linear regression. The mediating effect was tested via the bootstrap method in Process 4.1. *P* < 0.05 indicated statistical significance.

## Results

### Demographic profile of the subjects

We enrolled 190 elderly individuals, including 69 males and 121 females, and their average age was 81.49 ± 8.36 years. Table 1 presents more detailed information.

**Table 1:** Distribution of characteristics of participants (*N* = 190)

Project	Group	Number of persons	Percentage (%)
Age (years old)	60–69	18	9.5
	70–79	46	24.2
	80–89	98	51.6
	90 and above	28	14.7
Sex	Male	69	36.3
	Female	121	63.7
BMI	<18.5	27	14.2
	18.5–23.9	110	57.9
	≥24	53	27.9
Marital status	In marriage	73	38.4
	Not married	117	61.6
Education	Primary school and below	85	44.7
	Junior High School	44	23.2
	High School	43	22.6
	University and above	18	9.5
Monthly income (CNY)	<1000	26	13.7
	1000–1999	17	8.9
	2000–3999	45	23.7
	≥4000	102	53.7
disease type	Without	19	10.0
	1–2	115	60.5
	≥3	56	29.5
Walking assistance	Have	109	57.4
	Without	81	42.6
leisure activities	Have	105	55.3
	Without	85	44.7

*BMI *Body Mass Index, *CNY *Chinese Yuan (1 CNY = 0.137 USD)；Not married (including widowed, unmarried,divorced)

### Frailty status of old nursing home residents

The frailty prevalence of older nursing home residents was 34.74%, with a mean frailty score of 2.67 ± 1.03.

### Correlation analysis of frailty, sarcopenia, and loneliness in elderly nursing home residents

As revealed by Pearson correlation analysis, loneliness scores were positively related to frailty scores (*r* = 0.156, *P* < 0.05, 95% CI:0.014–0.292), sarcopenia scores were positively related to frailty scores (*r* = 0.512, *P* < 0.01, 95% CI:0.399–0.610), and loneliness scores were positively related to sarcopenia scores (*r* = 0.214, *P* < 0.01, 95%CI:0.074–0.346) among elderly nursing home residents (Table 2).

**Table 2:** Correlation analysis between loneliness, sarcopenia and frailty of the elderly in old-age care institutions

Project	Frailty score	Sarcopenia score	Loneliness score	
Frailty score	1	—	—	
Sarcopenia score	0.512*** (95% CI:0.399–0.610)	1	—	
Loneliness score	0.156* (95% CI:0.014–0.292)	0.214*** (95%CI:0.074–0.346)	1	

*P<0.05;**P<0.01;***P<0.001;CI:confidence interval

### Analysis of the mediating effects of sarcopenia on loneliness and frailty in nursing home elderly patients

**Fig. 1:**
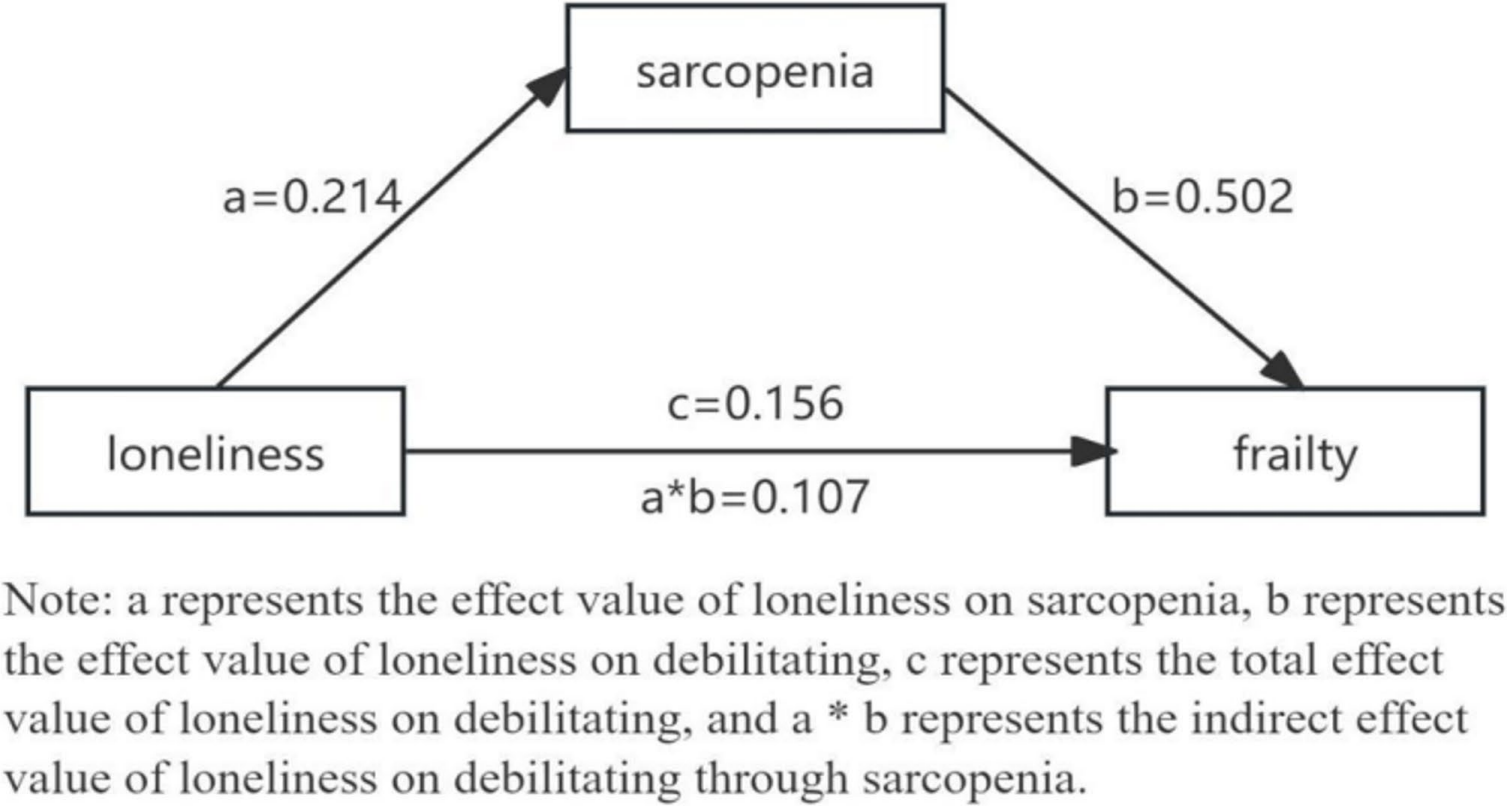
Mediation model and regression coefficient of sarcopenia between loneliness and frailty

Regression analysis was performed, with loneliness being an independent variable (X), sarcopenia being a mediator (M), and frailty being a dependent variable (Y). First, in the regression analysis with loneliness as the independent variable and frailty as the dependent variable, loneliness was found to significantly and positively predict frailty (c = 0.156, *P* < 0.05). Second, regression analysis with loneliness as the independent variable and sarcopenia as the dependent variable indicated that loneliness also significantly and positively predicted sarcopenia (a = 0.214, *P* < 0.01). Third, in the regression model with both loneliness and sarcopenia as independent variables and frailty as the dependent variable, the coefficient for sarcopenia was b = 0.502 (*P* < 0.01), while the coefficient for loneliness was reduced to c’ = 0.049 (*P* > 0.05). These results suggest that sarcopenia fully mediates the relationship between loneliness and frailty among elderly individuals residing in nursing homes. Refer to Table 3; Fig. 1 for details of the mediation model.

**Table 3:** Regression analysis between sarcopenia and loneliness on elderly frailty in old-age care homes

Predictive variable	Model 1		Model 2		Model 3	
	β	t	β	t	β	t
Loneliness	0.156	2.172**	0.214	3.002***	0.049	0.766*
Sarcopenia					0.502	7.813***
R^2^	0.024		0.046		0.265	
F	4.717		9.011		33.634	

Model 1: Frailty predicted by loneliness; Model 2: Sarcopenia predicted by loneliness; Model 3: Frailty predicted jointly by loneliness and sarcopenia. *P >0.05; **P < 0.05; ***P < 0.01.β:partial regression coefficient

### Bootstrap mediation analysis of sarcopenia on loneliness and frailty in nursing home elderly patients

The ability of sarcopenia to mediate the association of loneliness with frailty in elderly nursing home residents was assessed via the bootstrap method. Through the use of 5,000 bootstrap samples, we tested the indirect effect of loneliness on frailty via sarcopenia. 0 was not included in the 95% confidence interval of this indirect effect. After the sarcopenia variable was incorporated, loneliness did not exert an obvious direct effect on frailty (95% CI including 0), indicating that sarcopenia fully mediates the association of loneliness with frailty in nursing home-aged elderly individuals. The total effect of loneliness on frailty was 0.156. Sarcopenia had a mediating effect of 0.107, accounting for 68.59% of the overall effect. See Table 4.

**Table 4:** Bootstrap mediation effect test of sarcopenia between loneliness and frailty of the elderly in old-age care institutions

Project	Effect value	BootSE	95% CI	Proportion(%)
Total effect	0.156	0.072	0.014–0.299	—
Indirect effects	0.107	0.044	0.026–0.197	68.59%
Direct effect	0.049	0.064	-0.078–0.176	31.41%

## Discussion

### Current status of frailty among nursing home elderly individuals

This study found that the mean frailty score among elderly nursing home residents was 2.67 ± 1.03, with a frailty prevalence of 34.74%.The findings of this study are consistent with those reported by Zhao Xia et al. [17] (37.8%), but notably higher than those of Xu Weiwei et al. [18] (18.0%) and a large-scale survey involving 19,649 elderly individuals across 12 European countries (10.2%) [19]. This discrepancy may be attributed to the higher proportion of very old individuals and females in the current study’s sample. Previous research has identified advanced age and female gender as significant risk factors for frailty [4, 20]. This study’s findings indicate a relatively severe frailty status among elderly nursing home residents. These findings suggest that medical staff in nursing homes should focus on frailty screening and develop targeted interventions based on factors influencing frailty to improve the physical condition of elderly individuals.

### Correlation analysis of loneliness, sarcopenia, and frailty among elderly nursing homes

Our findings revealed that loneliness was positively related to frailty (*r* = 0.156, *P* < 0.05). In some studies, elderly individuals experiencing high levels of loneliness have an increased risk of frailty relative to those experiencing low levels of loneliness [21, 22]. Loneliness among the elderly is often linked to reduced social support and decreased social participation. Inadequate social support may hinder access to timely healthcare services, thereby elevating the risk of frailty [9]. A longitudinal study conducted among Dutch older adults found that individuals who were physically frail were more likely to experience loneliness in subsequent follow-up assessments [23]. This association may stem from the limitations in daily living activities commonly observed in physically frail individuals, which in turn restricts their social engagement [24]. Studies have indicated that augmenting social support and physical exercise and enhancing healthy behaviors can help decrease the incidence of frailty and help prevent loneliness [8, 9].

This study revealed that loneliness was positively related to sarcopenia (*r* = 0.214, *P* < 0.01). Loneliness not only affects the mental health of older people but can also induce inflammatory responses, leading to increased interleukin-6 and C-reactive protein levels, which are potential risk factors for sarcopenia [25]. Additionally, a state of chronic inflammation can inhibit the synthesis of muscle protein and accelerate the loss of muscle mass, thereby promoting the development of sarcopenia [26]. During the COVID-19 isolation period, reduced social contact led to a greater incidence of loneliness in elderly individuals, which in turn increased the risk of sarcopenia by affecting physical activity levels and nutritional intake in elderly individuals [10].

This study revealed that sarcopenia scores are positively correlated with frailty scores (*r* = 0.512, *P* < 0.01). Compared with their nonsarcopenic counterparts, individuals who develop sarcopenia have a 2-fold increased risk of frailty [5]. In patients with sarcopenia, decreased muscle mass, strength, and physical function can directly affect walking speed and grip strength [27]. The pathogenesis of sarcopenia is complex. Hormonal dysregulation, cardiomyocyte changes, and inflammatory responses can reduce muscle strength, limiting the physical activity of elderly individuals and leading to frailty [28]. Additionally, individuals experiencing frailty face an increased risk of sarcopenia [12]. Frailty and sarcopenia, both of which are age-related geriatric syndromes, significantly overlap in terms of “muscle strength and physical function”. Both can reduce muscle strength and mobility [5, 12, 29].

### The mediating effect of sarcopenia on the association between loneliness and frailty among old nursing home residents

We found that loneliness was correlated with frailty among older people. When sarcopenia was added to the regression equation, the regression coefficient for loneliness on frailty became insignificant (*P* > 0.05). Consequently, sarcopenia fully mediates the association of loneliness with frailty in nursing home-aged elderly individuals. Pan Chaoping’s [30] study also indicated that loneliness in elderly individuals is not a factor that independently predicts frailty. It may affect the health of older people through other pathways.

Further research has indicated that loneliness can decrease an individual’s motivation to engage in daily activities and is associated with sleep disturbances and malnutrition—factors known to contribute to the development of sarcopenia [10, 31–33]. Sarcopenia, in turn, is a key contributor to functional decline and frailty [22]. Evidence suggests that interventions such as vitamin D and protein supplementation, combined with resistance training, can enhance muscle quality and strength in elderly individuals affected by sarcopenia [34–36]. Therefore, targeting sarcopenia may serve as an effective strategy for preventing frailty among older adults in nursing homes. Loneliness has an important effect on sarcopenia and frailty in elderly individuals. However, compared with physical health issues, the psychological state of elderly individuals is often neglected [28]. Lonely elderly individuals are prone to engaging in health-damaging behaviors such as insufficient physical activity, smoking, and disordered sleep patterns, all of which are detrimental to improving frailty [37]. Therefore, when nursing home medical staff intervene to address frailty in elderly individuals, they should simultaneously monitor loneliness levels and sarcopenia status and actively conduct screenings and interventions.

### Limitations

This study has certain limitations. First, it surveyed only 190 elderly individuals from four nursing homes located in Guangzhou, Dongguan, and Zhanjiang in Guangdong Province, and Nanning in Guangxi Province. As a result, the representativeness of the sample is somewhat constrained. Future research should consider expanding the sample size and geographic scope. Second, due to the cross-sectional design of the study, causal relationships could not be established. Longitudinal studies are recommended to strengthen the scientific validity of the findings. Third, this study did not explore potential biases in depth. For example, social desirability bias may affect the accuracy of elderly self-reports on loneliness. Future research should use more complex designs to control for these biases.

## Conclusions

This study reveals significant correlations among loneliness, sarcopenia, and frailty. Sarcopenia is identified as a full mediator in the relationship between loneliness and frailty, providing theoretical guidance for interventions targeting frailty. The findings also underscore the critical role of healthcare professionals in nursing homes in addressing the psychological well-being of older adults by actively implementing measures for loneliness screening and intervention.

## Supplementary Information

Supplementary Material 1.

## Data Availability

The datasets used and analyzed in the current study are available from the corresponding author on reasonable request.

## Abbreviations

BMI: Body Mass Index
CI: Confidence interval
CNY: Chinese Yuan
UCLA Loneliness Scale: University of California, Los Angeles Loneliness Scale
SARC-F: Strength, Assistance with walking, Rise from a chair, Climb stairs and Falls
