# Supplementary material for: The mediating role of sarcopenia in the link between loneliness and frailty among nursing home residents: a cross-sectional study
Source: BMC Geriatr. 2025 Sep 26;25:722. doi: 10.1186/s12877-025-06342-5 (PMC12465528; doi:10.1186/s12877-025-06342-5)
Supplement: Supplementary file 1 — Supplementary Material 1. [file 12877_2025_6342_MOESM1_ESM.pdf]

CERTIFICATE OF ENGLISH EDITING

This is to certify that the manuscript entitled

The Mediating Role of Sarcopenia in the Link Between Loneliness and Frailty Among Nursing Home Residents: A Cross-Sectional Study.

By: Zhenfei Chen,Dongxing Zheng,Shangqing Wu,Jiaze Dai,Haiman Huang,Yu Yang .

commissioned to us has been carefully edited by a native English-speaking editor at Editeg. The grammar, spelling, and punctuation of the text have carefully been checked and corrected wherever required. We believe that the language of this paper has been considerably improved to meet academic standards. You may please contact us for further queries regarding the editing process.

LANGUAGE EDITING EDITEG

Date of issue
June 26, 2025

Disclaimer: The changes in the document may be accepted or rejected by the authors at their sole discretion after our editing. Therefore, Editeg would not be responsible for the revisions made to this document after our editing carried on **June 26, 2025**

Editeg Website:https://www.editeg.com

2000+ native English editors:https://www.editeg.com/
